# Consenting for contact? Linking electronic health records to a research register within psychosis services, a mixed method study

**DOI:** 10.1186/s12913-015-0858-4

**Published:** 2015-05-14

**Authors:** Dan Robotham, Simon Riches, Iain Perdue, Felicity Callard, Thomas Craig, Diana Rose, Til Wykes

**Affiliations:** Institute of Psychiatry, Psychology & Neuroscience, Kings College London, London, SE5 UK; Centre for Medical Humanities, Durham University, Caedmon Building, Leazes Road, Durham, DH1 UK

**Keywords:** Participation, Psychosis, Health records, Clinical trials, Research register, Secondary care, Informed consent

## Abstract

**Background:**

Research registers of potential participants linked to Electronic Health Records (EHRs) provide a basis for screening and identifying people suitable for studies. Such a system relies upon people joining the register and giving permission for their record to be used in this way. This study describes the process of training clinicians to explain EHR-linked research registers to service users, and to recruit them onto the register.

**Method:**

Training materials were developed for clinicians to help them describe the register to service users. These materials were based upon findings from focus groups reported elsewhere, they were then tested with 31 clinicians in early intervention psychosis services and each clinician discussed the register with service users on their caseload (n = 100 service users). Consultations were recorded and analysed in relation to their coverage of the training criteria. Service users also provided data on the acceptability of the process from their perspective. The content of clinicians’ explanations to service users was described, and then compared against the likelihood of service users joining the register. Interpretive statistics (t-test and Chi-Squared) were used to explore differences between consultations in which service users agreed to join the register, and consultations where they did not agree to join.

**Results:**

Service users appeared more likely to join the register if they felt control over what they signed up to, this necessitated understanding that they could decide when, how often, and by whom they were contacted, that joining the register did not automatically enlist them to future studies, and that they could change their mind in future. Clinicians’ explanations did not always include that researchers would be able to see the service users’ EHR. Service users often confused the idea of signing up to the register and signing up to studies themselves. Confidentiality was not well explained, but service users were not always concerned by confidentiality.

**Conclusion:**

EHR-linked research registers provide recruitment opportunities, and help service users to find out about research. Implementing these registers within mental health settings requires a trained clinical workforce and an informed service user population.

## Background

Recruiting participants to health research is essential in order to develop evidence-based treatments and therapies. However, clinical trials often fail to recruit sufficient numbers of participants [1-3] and so make the study scientifically unsound. Obstacles to recruitment are either ‘patient based’ or ‘clinician based’ [4]; for patients, these include additional efforts like travel, money, procedures, and worries about receiving placebo treatments; for clinicians they include lack of time, loss of authority, worries about patients and difficulty with the consent process.

One way to improve recruitment has been to create a register of people who are interested in research opportunities. Research registers have been shown to improve recruitment percentages and to lower recruitment resources [5]. Anyone can create or host a register, e.g., researchers, clinical teams, voluntary sector organisations, or patient/service user groups. Typically, researchers wishing to access a register would prepare an advert for recruitment (using wording approved by an Ethics Committee), which the host would then circulate. It is now possible to link a research register to information in the patient’s Electronic Health Record [6, 7]. Potential participants can then be identified before approaching them, with more specific detail, thus making it easier to assess potential participant eligibility.

Anonymous EHRs can already be used for research purposes without consent. One example is the Clinical Record Interactive Search (CRIS) within South London and Maudsley NHS Foundation Trust (SLaM) [8], which allows researchers to access data from over 200,000 EHRs, of which an estimated 37,000 records relate to active individuals. CRIS was developed with input from service users to improve its acceptability [9]. De-anonymising these records for the purposes of research recruitment may lead to complications in mental health settings due to the potentially sensitive nature of recorded information. Added complications may exist for people experiencing symptoms of psychosis; one study found that the idea of health records were least acceptable to people experiencing paranoia and delusions of conspiracy [10], and people with psychosis comprise a large proportion of the population using SLaM services.

Linking EHRs to a research register represents a shift from traditional recruitment methods in health and social care. Relationships between service users and researchers are usually brokered through clinician gate-keepers [11], who often manage busy caseloads, but who may lack the confidence to introduce research to people and/or answer questions that arise [12].

Following the development of a local EHR-linked research register [13], local service users and clinicians provided feedback on the process and identified barriers to implementation. The register was praised for its ability to improve research recruitment and promote autonomy, but there were concerns around the potential for coercion and the maintenance of confidentiality around sensitive information. Some clinicians also expressed wanting to retain involvement in inviting service users to join the register [14]. This resulted in the development of standardised training materials to help clinicians explain the EHR-linked register to service users on their caseload. This included a checklist of items that arose from the focus group. The current study reports the testing and continued development of these training materials within services for people with psychosis. We also gained feedback from service users on the acceptability of clinicians’ explanations.

### Aims

We aimed to develop routine ways in which the EHR-linked research register could be explained to people who use psychosis services, and to ascertain the importance of the explanation in encouraging service users to sign up.

## Methods

### Design

This was an iterative, mixed method study to examine how clinicians explained the EHR-linked register after being trained and given a checklist of items to mention. Service users were involved in developing, designing, advising on, and carrying out the research. Qualitative analyses were conducted throughout the study to inform the continued development of the training materials. To ascertain how the EHR-linked register was being explained, consultations between clinicians and service users were recorded and analysed using a checklist of information items. Items were scored ‘1’ if they appeared in clinicians’ explanation and ‘0’ if they did not. This was analysed with reference to the service users’ decision to join the register.

### Sample and recruitment

Participants were recruited from early intervention services for people with psychosis. These services are currently a key area for research and so were chosen for contextual reasons. Staff participants (n = 31) were recruited from all five early intervention teams in SLaM. Those recruited were predominantly care coordinators and clinical psychologists. Between them, clinicians identified 100 service users with whom they had an existing care relationship and who they considered appropriate to invite for a recorded discussion about the register. Participating service users were aged between 18 and 47, and had capacity to consent. All participants were given information about the study and provided written informed consent. The study was approved by the National Research Ethics Service, London - Dulwich (reference: 11/LO/1255).

### Procedure

Written informed consent was gained from both the clinician and service user.

There are three elements:

1. ***Training:*** The research team trained participating clinicians on how to explain the EHR-linked register to service users, this included reference to the checklist of items that was developed following prior focus groups with service users and clinicians [14]. The standardised training was based on previous consultations and following the analysis of the first 25 consultations it was adapted to emphasise three elements: (i) the benefits of research, (ii) examples of research that might be available and (iii) the difference between joining the register and joining future studies.
2. ***The ‘joining the register’ conversation:*** The clinician explained the register to the service user and the conversation was audio recorded.
3. ***Service user interview:*** This was carried out immediately after the register conversation. The service user was interviewed and asked to summarise the key points from their consultation. They also rated on a five point Likert-scale; (1) whether the idea of being contactable for research had been explained to them, (2) whether they felt able to ask questions, (3) whether they had enough time to make a decision, (4) whether they felt pressurised to say yes, and (5) whether they were worried about confidentiality.

### Data analysis

Initial analyses were conducted after data had been collected from 25 consultations, two independent researchers conducted thematic analysis using NVivo using the coding framework derived from the checklist. These analyses were only to inform training. After data collection was completed, three researchers independently analysed all 100 consultation transcripts (including those 25 analysed previously) in NVivo, in relation to the checklist. Each consultation transcript was checked against the items in the revised checklist. This was subjected to inter-rater reliability testing using Kappa in Stata. Descriptive and interpretive statistics (using SPSS) were used to analyse whether service users’ sign-up rates were related to the content of clinicians’ explanations, and to ascertain differences across service users’ responses to the Likert scale questions.

## Results

### Participants

The mean age of service user participants was 26 (range 18–47), and the majority came from black and minority ethnic groups. Most had engaged with mental health services for less than 5 years. The majority of the staff participants were white, female, and employed as care co-ordinators or psychologists. Participant demographics are shown in Table 1.

**Table 1:** Participant demographic data

Service users	No. & % of sample	Staff	No. & % of sample	
Gender		Gender		
Male	68	Male	12	38.7
Female	32	Female	19	61.3
Ethnicity		Ethnicity		
White	30	White	23	74.2
Black/Black British	50	Black/Black British	4	12.9
Asian/Asian British	1	Asian/Asian British	4	12.9
Mixed heritage	7			
Other	12			
Diagnosis		Staff role		
Psychosis/schizophrenia	67	Care co-ordinator	20	64.4
Bipolar affective disorder	13	Nurse	1	3.2
Depression/anxiety	7	Psychologist	4	12.9
At risk mental state	6	Team manager	2	6.5
Personality disorder	1	Psychiatrist	2	6.5
Other	3	Occupational therapist	2	6.5
Not known	3			
Time with team		Team		
Less than 6 months	37	Team A	11	35.5
6-12 months	18	Team B	12	38.7
+1 year	40	Team C	5	16.1
+5 years	5	Team D	2	6.5
		Team E	1	3.2
**Years in mental health services**				
0-5	88			
6-10	12			
TOTAL	100		31	

### Analysis of consultations

On average, consultations between service users and clinicians lasted an average of six and a half minutes (min = 2, max = 22), with the majority (66 %) lasting between 4 and 8 min. Table 2 shows the number of consultations (total n = 100) in which clinicians mentioned the 18 items, and the number of times they were either mentioned by service users throughout the course of the consultation, or recalled during summaries given to the researcher.

**Table 2:** Items required for explaining an EHR-linked research register, and how often they occur

Item #	Item	Clinician mentioned (Total potential n 100)	SU mentioned in consultation or summary to researcher (Total potential n 100)
1	Having a health record	77	0
2	Having an EHR	66	0
3	Confidentiality of EHR	28	0
4	Benefits of research	80	15
5	Types of research	54	12
6	Personalised example of research	48	4
7	Researchers have been ‘approved’	52	6
8	Researchers’ confidentiality	37	12
9	Researchers will identify you from EHR	62	12
10	Researchers may contact you in future	94	44
11	The register is voluntary	77	23
12*	Future studies are voluntary	62	17
13*	Service user can change their mind	69	23
14	Decision won’t affect care	32	2
15*	Can stipulate, i.e., what/when/how contacted	77	18
16	Ask whether they wish to join the register	84	0
17	Questions and concerns	67	0
18	Who to contact for further information	58	0

*significant differences between ‘yes’ and ‘no/undecided’ samples

When explaining the EHR-linked register to service users, clinicians only mentioned EHRs in 66 % of consultations. Whilst most consultations contained reference to the fact that researchers might contact them in future to invite service users into research studies (94 %), clinicians less often mentioned the fact that this contact would be based upon researchers accessing their EHR (62 %). Service users’ comments reflect this during the consultation and/or their summary to the researcher; 44 % recalled that researchers would contact them in future, but only 12 % recalled that this would be based upon information in their EHR. Service users’ percentages should not be compared against clinicians’ percentages, since service users may have understood aspects without verbalising them to the researcher.

The most common example of service user misunderstanding related to confusing the process of signing up to the register with the process of signing up to studies. Clinicians only made this distinction in 62 % of the consultations. 15 % of service users voiced a misunderstanding to this effect, for example, asking questions such as “is the research starting now?”

Group comparisons identified differences between the service users who joined the register (n = 86), and those who refused to join (n = 14). Chi-Squared tests (two-sided) were used to test differences in service users’ responses based upon what clinicians had mentioned to them, as listed in Table 2. Significant differences in whether service users joined the register were found to relate to whether clinicians had explained three items; that future studies were voluntary (*χ*^2^ = 4.38, df = 1, p = .036, C = .95), that the service user can change their mind (*χ*^2^ = 5.71, df = 1, p = .017, C = .95), that the service user can stipulate aspects of the sign up process (*χ*^2^ = 5.32, df = 1, p = .021, C = .95).

Confidentiality was another factor that related to service users’ willingness to sign up, although only 28 % of consultations included reference to the confidentiality of EHRs, and only 37 % described the confidentiality of researchers accessing EHRs. Most service users did not report feeling worried about confidentiality (in response to Likert question, Mean = 2.3, SD = 1.4), but those that did not join the register reported greater concerns (Mean = 3.9, SD = 1.3) than those who joined (Mean = 2, SD = 1.3). This difference was also significant (t = −5.25, df = 98, p = .000, C = .95).

According to the Likert scale responses for this study, most service users did not feel pressure to sign up to the register (Mean 1.48, SD = 0.88), they felt they had enough time to make a decision (Mean 4.29, SD = 1.05), and felt able to ask questions (Mean 4.64, SD = 0.72).

The revised training improved clinicians’ explanations. A comparison of the first 25 interviews and the remaining 75 showed that some parts of the item explanation checklist were more likely to be mentioned; (i) explaining EHRs (73 % Vs 36 %, *χ*^2^ = 11.34, df = 1, p = .001, C = .95), (ii) explaining the benefits of research (87 % Vs 60 %, *χ*^2^ = 8.33, df = 1, p = .006, C = .95), (iii) that researchers would contact service users in future (99 % Vs 80 %, *χ*^2^ = 11.58, df = 1, p = .003, C = .95), (iv) that such studies would be voluntary (67 % Vs 44 %, *χ*^2^ = 4.05, df = 1, p = .039, C = .95) and finally (v) service users were explicitly asked whether they wanted to join the register (95 % Vs 56 %, *χ*^2^ = 21.2, df = 1, p = .001, C = .95).

## Discussion

The findings demonstrate that, in explaining the EHR-linked research register, clinicians must clarify that service users have a choice over the following; contact preferences, rights to leave the register, rights to refuse participation in research. One common misunderstanding seems to have an impact on service users’ perceived freedom; the conflation of joining the register and of joining research studies. Although there was significant improvement in how this was explained after the training materials were adapted, still only two thirds of clinicians distinguished between these two concepts when talking to service users.

Despite some gaps in clinicians’ explanations, most service users did not feel pressurised to join the register. The majority of those who were asked to join the SLaM EHR-linked register have done so. At the time of writing (01.06.2014), 3764 of 5230 people had joined (72 %). Specifically within psychosis services, 787 of 1207 people have joined (65 %) which is a little lower than our group but this is with clinicians who had not necessarily had the more rigorous training that we provided in this study.

Our results show that clinicians did not always explain how the service users’ EHRs was to be used; either regarding confidentiality or regarding how they would be accessed by researchers. This reflects previous research, which has suggested that EHRs are not well understood [15], and their accuracy is contested [16]. One problem is that an EHR-linked research register necessitates the use of identifiable data for research purposes, but even the use of anonymous NHS data has met with concerns, 45 % of the population remain unaware of NHS plans to make their anonymous records accessible for research purposes [17]. The transparency of online mental health information in EHRs is controversial with 66 % of doctors and 73 % of the public thinking that such ‘sensitive information’ should never be made accessible online [18].

The South London and Maudsley NHS Foundation Trust is developing a system through which selected parts of the clinical record, such as the care plan, can be shared between service users and clinicians (the myhealthlocker™ system [19]). In future, such a system might allow the service user to manage the EHR information and to choose which parts they wish to share on a research register. This may help service users feel as though they are participating in building an accurate health care record.

One way in which service users could feel more control over joining an EHR-linked register is to join the register directly, rather than waiting for clinicians to invite them. This procedure aligns with the messages of public campaigns such as the National Institute of Health Research’s ‘OK to Ask’ in the UK, which encourage all patients to ask healthcare professionals about clinical trial opportunities. Those elements from the study that aid explanations can be transferred from a clinical context into a NHS patient campaigning context. In SLaM, this is already underway. Service users can apply to join the register, and a notification of this application is sent to the individual’s care team.

There are some strengths and limitations to the present study. One strength was a high level of service user involvement both in the development of the training protocol, in the design of the study and in the collection of data. It may be that the sample of service user participants (who were chosen by clinicians) might be biased towards those with a greater understanding and willingness to take part in research which is why we had higher sign up rates than in the rest of the local services. But an alternative explanation might be our more rigorous training programme. Changes in the programme seemed to have an effect on the items that service users wanted to hear, suggesting that training may improve the understanding and hence lead to higher agreement rates amongst service users. A further limitation is that we analysed the content of clinicians’ explanations, not the narrative clarity. But even with both these potential limitations we were able to detect that some categories of information seem to be linked to decisions to accept the research register.

## Conclusions

EHR-linked research registers have the potential to revolutionise clinical research, aiding researchers with recruitment, allowing patient populations (including mental health service users) to find out about relevant research projects, and reducing the burden of clinicians who are asked by researchers to recruit people from their caseload. We have provided some evidence that such a register is possible within perhaps the most challenging context – in psychosis services. The next phase will be to see whether the register is helpful to researchers and acceptable to registered service users.

## Abbreviations

BME: Black and Minority Ethnic
CRIS: Clinical Record Interactive Search
EHR: Electronic Health Record
NHS: National Health Service
SLaM: South London and Maudsley NHS Foundation Trust
SPSS: Statistical Package for the Social Sciences
